# Self-assessment of auditory verbal hallucinations in schizophrenia; validation of a digital device

**DOI:** 10.1192/j.eurpsy.2023.1047

**Published:** 2023-07-19

**Authors:** S. Dollfus, F. Letourneur, L. Metivier, V. Moulier, M. Rotharmel

**Affiliations:** 1 University Caen Normandie; 2 University Caen Normandie, Caen; 3 CH du Rouvray, Sotteville les Rouen, France

## Abstract

**Introduction:**

Auditory verbal hallucinations (AVH) are experienced by approximately 70% of patients with schizophrenia. At the present time, there are no self-evaluation scales for auditory verbal hallucinations. They would allow the patient to self-assess their hallucinations when they occur, taking into account the great variability over time. Moreover, self-assessment allows the patients to better recognize their symptoms and to be more engaged in their treatment. In this context, we have developed a digital device (MIMO) allowing the patient to self-evaluate his/her AVH and to declare his/her hallucinatory crisis at any time. This device contains a self-assessment of auditory verbal hallucinations (SAVH) with 13 questions, 9 of which concern the frequency, the severity, the content and the impact of hallucinations. These 9 questions are rated on a 5-point scale ranging from 0 (absent) to 5 (severe). The patients and practitioners can have an online feedback on the scores as well as on their temporal changes.

**Objectives:**

The aim of this study was to validate the SAVH scale as well the digital tool, to demonstrate the acceptability by the patients and to prove the feasibility in using such a digital device (mobile phone or tablet).

**Methods:**

Forty one patients with schizophrenia or schizoaffective disorder (DSM-5) with AVH loaded this application on their own mobile or on a loaned one. AVH was also assessed with the Auditory Hallucination Rating Scale associated with the Brief Psychiatric Rating Scale and the self-assessment of insight. Moreover, a questionnaire included a visual analogic scale on the global satisfaction of the device scoring from 0 (“Not at all satisfied”) to 10 (“Very satisfied”) and 22 questions concerning the conditions of use, the acceptability and the content of the app, its impact on mental health, and questions related to the declaration of hallucinatory crisis. Moreover, statistical analyses were carried out testing internal, external and construct validities of the SAVH.

**Results:**

56.1% and 36.6% of patients found the app to be easy and very easy to use, respectively. 61% and 29.3% of the patients considered that the questions were respectively rather adapted and very adapted to the evaluation of auditory hallucinations. 46.3% of patients found the questions quite easy to understand. The majority of patients felt that the MIMO app could be useful to them. Overall satisfaction was 8.073+-3.8 indicating very good overall patient satisfaction of the app. Statistical tests revealed significant convergence and divergence validities as well as good internal consistency of the SAVH.

**Conclusions:**

This study demonstrated good psychometric properties of the SAVH and very good acceptability of this kind of assessment by digital device in patients with schizophrenia. Such a device can be quite useful to assess the efficacy of the treatment of AVH and to increase the patient’s empowerment.

**Disclosure of Interest:**

None Declared

